# REFRAMING GEROSCIENCE TO IMPROVE OUTCOMES IN PEOPLE LIVING WITH CHRONIC INFECTION

**DOI:** 10.1093/geroni/igae098.0320

**Published:** 2024-12-31

**Authors:** Monty Montano

**Affiliations:** Harvard Medical School, Boston, Massachusetts, United States

## Abstract

This talk will highlight recent advancements in aging science as applied to individuals living with chronic conditions associated with infections, such as HIV-1 and SARS-CoV-2. The discussion will focus on aging biomarkers and health trajectories in individuals with chronic morbidities related to infection. Additionally, the talk will address the importance of considering both physiological and holistic outcomes, taking into account allostatic load and the life history of stressors, both biological and psychosocial in strategies designed to improve quality of life.

